# Commentary: Chronotype, circadian rhythm, and psychiatric disorders: recent evidence and potential mechanisms

**DOI:** 10.3389/fnins.2024.1470026

**Published:** 2024-11-01

**Authors:** José Francisco López-Gil, Juan Ramón Barrada

**Affiliations:** ^1^One Health Research Group, Universidad de Las Américas, Quito, Ecuador; ^2^Department of Psychology and Sociology, University of Zaragoza, Zaragoza, Spain

**Keywords:** chronotype, disordered eating, eating disorders, chronobiology discipline, circadian rhythm disorders

## Introduction

This commentary tries to express an appreciation for the insightful article “Chronotype, circadian rhythm, and psychiatric disorders: recent evidence and potential mechanisms” by Zou et al. (2022), published in Frontiers in Neuroscience. The comprehensive review provided a nuanced understanding of how chronotype is related to various psychiatric disorders, offering a valuable resource for both researchers and clinicians in the field.

## Discussion on circadian mechanisms and circadian typology

Circadian mechanisms and circadian typology are related but distinct concepts. Circadian mechanisms refer to the biological processes that regulate the body's internal clock, governing physiological functions like sleep, metabolism, and hormonal cycles (Ayyar and Sukumaran, 2021). On the other hand, circadian typology refers to individual differences in circadian preferences, such as being a morning person, an evening person, or somewhere in between (Roenneberg et al., 2007). Future research on circadian mechanisms and circadian typology is essential for advancing our understanding and treatment of psychiatric disorders, including eating disorders (Kandeger et al., 2021; Wilcox et al., 2024). As we delve deeper into how variations in circadian rhythms—such as differences in sleep-wake cycles, hormonal secretion patterns, and core body temperature rhythms—affect vulnerability to these disorders, new opportunities arise for developing more precise and individualized interventions. By identifying how specific circadian typologies (morning, intermediate, and evening types) align or misalign with these biological rhythms, researchers, and clinicians can create targeted prevention and treatment strategies that address the unique circadian characteristics of each individual. This personalized approach has the potential to significantly enhance therapeutic outcomes and provides a more accurate framework for addressing the complex relationship between circadian biology and mental health in the future.

## Discussion on evening chronotype and eating disorders

More concretely, the discussion on the association between evening chronotype and eating disorders was interesting. Zou et al. (2022) highlighted that the evening chronotype is significantly associated with eating disorders (which included, according to those researchers, rumination disorder, avoidant/restrictive food intake disorder, anorexia nervosa, bulimia nervosa, and binge eating disorder), with most studies focusing on binge eating disorder. However, this commentary tries to add some complementary information regarding this association, which might enrich the discussion further.

Zou et al. (2022) stated that Rodríguez-Cortés et al. (2022) found evidence linking evening chronotype to eating disorders in children and adolescents. However, in Rodríguez-Cortés et al. (2022)'s meta-analysis it was indicated that “no articles were found that addressed the chronotype of children and/or adolescents with typical eating disorders, i.e., anorexia nervosa, bulimia nervosa, or binge eating disorder”. Furthermore, a recent study by Wilcox et al. (2024) suggests that anorexia nervosa may actually align with a morning chronotype, which stands in contrast to the evening chronotypes typically associated with most other psychiatric disorders.

On the other hand, Rodríguez-Cortés et al. (2022) concluded that children and adolescents with an evening chronotype were more predisposed to food addiction (FA) and night eating syndrome (NES). It is supriginly because same authors indicated the study by Najem et al. (2020) did not find a direct relationship between chronotype and food addiction (FA), but evening-type individuals were found to have higher stress levels (*p* = 0.010). Regardless, none of those problematic eating behaviors were among listed as eating disorders by Zou et al. (2022).

Several points should be considered with the review of Rodríguez-Cortés et al. (2022) referenced by Zou et al. (2022). For the relation between chronotype and FA, Rodríguez-Cortés et al. (2022) only included a single study. Yet, surprisingly, as Rodríguez-Cortés et al. (2022) note, in that study no association was found between chronotype and FA (although it was associated with higher stress levels), which contradicts their overall conclusions. For chronotype and NES, two studies. Of those three studies, participants of two were university students (Kandeger et al., 2018: *M*_age_ = 21.1 years; Najem et al., 2020: *M*_age_ = 20.2 years). That is, the majority of samples included are not of children or adolescents.

In addition, further theoretical considerations can be made discussing the categorization of FA and NES as eating disorders.

## Clarification on FA and NES

FA and NES are abnormal eating patterns. FA is characterized by a compulsive relationship with food, which often involves cravings and a loss of control (Meule and Gearhardt, 2014). There is evidence that FA may represent a distinct phenomenon from established eating disorders (Gordon et al., 2018; Hauck et al., 2020). FA is not currently included as specific eating disorders as defined by standard diagnostic tools, such as those in the Diagnostic and Statistical Manual of Mental Disorders, Fifth Edition (DSM-5-TR) (American Psychiatric Association, 2022). Regarding NES, it involves nocturnal eating episodes and disrupted sleep patterns (Kaur et al., 2022). In the current DSM edition NES is classified as an Other Specified Feeding or Eating Disorder, that is, NES is not listed among the “core” eating disorders. Both conditions share some features with eating disorders, but they also exhibit distinct behavioral and psychological profiles (Yassibaş et al., 2024).

Parents, guardians and healthcare providers should not only be aware of diagnosed eating disorders but also of the symptoms of disordered eating. These symptoms include behaviors such as weight-loss dieting, overeating episodes, self-induced vomiting, excessive exercise, or the use of laxatives or diuretics (Toni et al., 2017). It is important to distinguish disordered eating from eating disorders (Quick et al., 2013), especially for children and adolescents, as the term disordered eating is often used to describe and identify some of the different eating behaviors that do not necessarily meet the diagnostic criteria for an eating disorder and therefore cannot be classified as eating disorders *per se* (Pennesi and Wade, 2016). In this sense, a previous systematic review and meta-analysis found that approximately 22% of children and adolescents exhibited disordered eating behaviors, with a higher prevalence in girls, older adolescents, and those with higher body mass index (López-Gil et al., 2023). While these behaviors may not warrant a clinical diagnosis of an eating disorder, they can still have negative outcomes related to eating disorders and obesity in adolescents (Neumark-Sztainer et al., 2006). Despite their impact on health being often minimized, disordered eating should be closely evaluated because it can evolve into eating disorders (Toni et al., 2017).

Considering this, it may be beneficial to view FA and NES as conditions that overlap with those eating disorders that have attracted more research, but also warrant separate investigation and categorization. This distinction is crucial for developing targeted interventions and treatment strategies that address the specific needs of individuals with these conditions. If evening chronotype's increased susceptibility to these eating behaviors in children and adolescents clearly need further research, as current evidence is limited (more limited than acknowledge by Rodríguez-Cortés et al., 2022 and Zou et al., 2022). It may be influenced by various factors, including circadian misalignment, stress, and difficulties with emotional regulation. Understanding the underlying mechanisms that drive these associations can provide valuable insights into effective management approaches.

## Effects of development and lifestyle on circadian mechanisms in youth

Childhood and adolescence are critical periods for brain development, marked by significant maturation of neural circuits and the hypothalamus, which regulates circadian rhythms (Logan and McClung, 2019). The brain's high neuroplasticity during these stages influences circadian regulation, affecting sleep-wake cycles and hormonal rhythms (Van Drunen and Eckel-Mahan, 2023). Modern lifestyles, including increased screen time, irregular sleep patterns, and late-night eating, can disrupt circadian mechanisms and lead to misalignment of internal clocks (Potter et al., 2016). These disruptions can impact cognitive function, emotional regulation, and overall health (Pifer et al., 2024). Understanding these effects is crucial for developing interventions to support optimal circadian rhythm alignment and health during these developmental stages.

## Conclusions

In conclusion, the contribution by Zou et al. (2022) to the understanding of chronotype and psychiatric disorders is meaningful. Their review highlights the importance of considering individual differences in chronotype when addressing mental health issues. The additional perspective on distinguishing “core” eating disorders (e.g., anorexia, bulimia, and binge eating) from FA and NES aims to complement their findings and encourage further research in this area. Future publications that continue to advance knowledge in this critical field are eagerly anticipated.

## References

[B1] American Psychiatric Association (2022). Diagnostic and Statistical Manual of Mental Disorders: DSM-5-TRTM (Fifth edition, text revision). New York: American Psychiatric AssociationPublishing. 10.1176/appi.books.978089042578738300502

[B2] Ayyar V. S. Sukumaran S. (2021). Circadian rhythms: Influence on physiology, pharmacology, and therapeutic interventions. J. Pharmacokinet. Pharmacodyn. 48, 321–338. 10.1007/s10928-021-09751-2 33797011 PMC8015932

[B3] Gordon E. Ariel-Donges A. Bauman V. Merlo L. (2018). What is the evidence for “food addiction?” A systematic review. Nutrients 10:477. 10.3390/nu10040477 29649120 PMC5946262

[B4] Hauck C. Cook B. Ellrott T. (2020). Food addiction, eating addiction and eating disorders. Proc. Nutr. Soc. 79, 103–112. 10.1017/S0029665119001162 31744566

[B5] Kandeger A. Egilmez U. Sayin A. A. Selvi Y. (2018). The relationship between night eating symptoms and disordered eating attitudes via insomnia and chronotype differences. Psychiatry Res. 268, 354–357. 10.1016/j.psychres.2018.08.003 30098543

[B6] Kandeger A. Egilmez Ü. Selvi Y. (2021). Feeding and eating disorders in the context of circadian rhythms. Alpha Psychiat. 22, 278–284. 10.5152/alphapsychiatry.2021.21151 PMC9685668 36448007

[B7] Kaur J. Dang A. B. Gan J. An Z. Krug I. (2022). Night eating syndrome in patients with obesity and binge eating disorder: a systematic review. Front. Psychol. 12:766827. 10.3389/fpsyg.2021.766827 35069340 PMC8766715

[B8] Logan R. W. McClung C. A. (2019). Rhythms of life: Circadian disruption and brain disorders across the lifespan. Nat. Rev. Neurosci. 20, 49–65. 10.1038/s41583-018-0088-y 30459365 PMC6338075

[B9] López-Gil J. F. García-Hermoso A. Smith L. Firth J. Trott M. Mesas A. E.. (2023). Global proportion of disordered eating in children and adolescents: a systematic review and meta-analysis. JAMA Pediatr. 177, 363–372. 10.1001/jamapediatrics.2022.5848 36806880 PMC9941974

[B10] Meule A. Gearhardt A. (2014). Food addiction in the light of DSM-5. Nutrients 6, 3653–3671. 10.3390/nu6093653 25230209 PMC4179181

[B11] Najem J. Saber M. Aoun C. El Osta N. Papazian T. Khabbaz L. R. (2020). Prevalence of food addiction and association with stress, sleep quality and chronotype: a cross-sectional survey among university students. Clin. Nutr. 39, 533–539. 10.1016/j.clnu.2019.02.038 30878156

[B12] Neumark-Sztainer D. Wall M. Guo J. Story M. Haines J. Eisenberg M. (2006). Obesity, disordered eating, and eating disorders in a longitudinal study of adolescents: how do dieters fare 5 years later? J. Am. Diet. Assoc. 106, 559–568. 10.1016/j.jada.2006.01.003 16567152

[B13] Pennesi J.-L. Wade T. D. (2016). A systematic review of the existing models of disordered eating: do they inform the development of effective interventions? Clin. Psychol. Rev. 43, 175–192. 10.1016/j.cpr.2015.12.004 26781985

[B14] Pifer G. C. Ferrara N. C. Kwapis J. L. (2024). Long-lasting effects of disturbing the circadian rhythm or sleep in adolescence. Brain Res. Bull. 213:110978. 10.1016/j.brainresbull.2024.110978 38759704 PMC11197883

[B15] Potter G. D. M. Skene D. J. Arendt J. Cade J. E. Grant P. J. Hardie L. J. (2016). Circadian rhythm and sleep disruption: causes, metabolic consequences, and countermeasures. Endocr. Rev. 37, 584–608. 10.1210/er.2016-1083 27763782 PMC5142605

[B16] Quick V. M. Byrd-Bredbenner C. Neumark-Sztainer D. (2013). Chronic illness and disordered eating: a discussion of the literature. Adv. Nutr. 4, 277–286. 10.3945/an.112.003608 23674793 PMC3650496

[B17] Rodríguez-Cortés F. J. Morales-Cané I. Rodríguez-Muñoz P. M. Cappadona R. De Giorgi A. Manfredini R.. (2022). Individual circadian preference, eating disorders and obesity in children and adolescents: a dangerous liaison? A systematic review and a meta-analysis. Children 9:167. 10.3390/children9020167 35204888 PMC8870066

[B18] Roenneberg T. Kuehnle T. Juda M. Kantermann T. Allebrandt K. Gordijn M.. (2007). Epidemiology of the human circadian clock. Sleep Med. Rev. 11, 429–438. 10.1016/j.smrv.2007.07.005 17936039

[B19] Toni G. Berioli M. Cerquiglini L. Ceccarini G. Grohmann U. Principi N.. (2017). Eating disorders and disordered eating symptoms in adolescents with type 1 diabetes. Nutrients 9:906. 10.3390/nu9080906 28825608 PMC5579699

[B20] Van Drunen R. Eckel-Mahan K. (2023). Circadian rhythms as modulators of brain health during development and throughout aging. Front. Neural Circ. 16:1059229. 10.3389/fncir.2022.1059229 36741032 PMC9893507

[B21] Wilcox H. Paz V. Saxena R. Winkelman J. W. Garfield V. Dashti H. S. (2024). The role of circadian rhythms and sleep in anorexia nervosa. JAMA Netw. Open 7:e2350358. 10.1001/jamanetworkopen.2023.50358 38175645 PMC10767597

[B22] Yassibaş E. Bölükbaşi H. Turan I. E. Demirel A. M. Gürler E. (2024). Hedonic hunger, food addiction, and night eating syndrome triangle in adolescents and its relationship with body mass index. J. Eating Disor. 12:25. 10.1186/s40337-024-00980-7 38336784 PMC10854182

[B23] Zou H. Zhou H. Yan R. Yao Z. Lu Q. (2022). Chronotype, circadian rhythm, and psychiatric disorders: Recent evidence and potential mechanisms. Front. Neurosci. 16:811771. 10.3389/fnins.2022.811771 36033630 PMC9399511

